# Rhabdomyolysis in older adults: outcomes and prognostic factors

**DOI:** 10.1186/s12877-023-04620-8

**Published:** 2024-01-11

**Authors:** Anne-Gaëlle Morin, Dominique Somme, Aline Corvol

**Affiliations:** 1 Geriatric Department, Univ Rennes, CHU Rennes, Rennes, F-35000 France; 2 Univ Rennes, EHESP, CNRS, Inserm, Arènes - UMR 6051, RSMS - U 1309, Rennes, F-35000 France; 3 CHU Pontchaillou, 2 Rue Henri le Guilloux, Rennes, 35000 France

**Keywords:** Rhabdomyolysis, Creatine kinase, Prognosis factor, Mortality rate, Older adult

## Abstract

**Background:**

Rhabdomyolysis is a common condition in older adults, often associated with falls. However, prognostic factors for rhabdomyolysis have mainly been studied in middle-aged populations.

**Objective:**

To test the hypothesis that age influences rhabdomyolysis prognostic factors.

**Methods:**

This retrospective single-center observational study included all patients with a creatine kinase (CK) level greater than five times normal, admitted to Rennes University Hospital between 2013 and 2019. The primary endpoint was 30-day in-hospital mortality rate.

**Results:**

343 patients were included (median age: 75 years). The mean peak CK was 21,825 IU/L. Acute renal failure occurred in 57.7% of the cases. For patients aged 70 years and over, the main etiology was prolonged immobilization after a fall. The 30-day in-hospital mortality rate was 10.5% (23 deaths). The Charlson score, number of medications and CK and creatinine levels varied according to age. Multivariate analysis showed age to be a factor that was associated, although not proportionally, with 30-day in-hospital mortality.

**Conclusion:**

Factors influencing rhabdomyolysis severity were not randomly distributed according to age. The term rhabdomyolysis encompasses various clinical realities and is associated with different mechanisms. More research is needed to better understand the physio-pathological and prognostic factors of rhabdomyolysis, especially in older adults.

## Introduction

Rhabdomyolysis is a clinic-biological entity in which striated muscle cells break down and release their contents into the bloodstream. The diagnosis is based on clinical and biological symptoms such as muscle pain, dark-tea colored urine, myoglobinuria and excessive potassium levels. It is confirmed by increased creatine kinase (CK) levels [1, 2]. The most commonly used threshold of CK for diagnosis is usually 1000 UI/l or five times the upper limit of normal levels [2]. Pathophysiological studies of this condition have shown striated muscle cells necrosis, through either direct injury (trauma/prolonged compression) or adenosine triphosphate (ATP) depletion, leading to a sudden increase in intracellular calcium. Striated muscle cells necrosis leads to the release of their contents into the serum (potassium, calcium, sodium, phosphorus, myoglobin, and CK). Precipitation of myoglobin in the renal tubules and vasoconstriction secondary to hypovolemia can lead to renal failure of varying severity, sometimes fatal [2].

Different etiologies have been described, and these can be grouped according to their mechanisms: hypoxic, traumatic, chemical, or biological. In adults, the most frequent causes are traumatic, iatrogenic, and infectious [3, 4]. Prolonged immobilization, usually after a fall, is the most common cause of rhabdomyolysis in older adults. Its occurrence increases with age and comorbidities [5]. In France, approximately 450,000 people over 65 years of age experience a fall each year, resulting in more than 9,000 deaths in 2013 [6]. A multicenter French study on 1467 subjects found that 14% of patients aged 65 years or more, hospitalized for an injury caused by a fall, had rhabdomyolysis [7].

An American study [8] proposed a composite score predictive of the risk of acute renal failure and mortality linked to rhabdomyolysis. It included initial CK level, the presence of acute renal failure, etiology, age, and values of phosphate, calcium, and bicarbonate. This approach was supported by subsequent studies [9] confirming that the composite score better predicted complications than the initial CK level alone. However, these studies are based on data obtained in middle-aged patients (median age: 50.7 years). We found few studies that looked at prognostic factors according to age.

Only two studies on rhabdomyolysis have focused specifically on older adults. The first is an American retrospective study in 2017 [10], focusing on patients (n = 167) aged 65 or older. Three prognostic factors for rhabdomyolysis were identified: polypharmacy, glomerular filtration rate (GFR) < 30 ml/min, and occurrence of acute renal failure. The second is also a 2017 retrospective study, conducted in Spain on 133 patients aged 65 years or older [11]. It showed a negative influence of creatinine levels, delirium, and acute brain injury on prognosis.

The majority (73%) of patients in the American study were of African-American descent, and only 20% of patients were Caucasian, which is not representative of the French population. The two aforementioned studies specifically focused on adults aged 65 years or older, and both showed the onset of acute renal failure as a poor prognosis indicator. These studies were based on a limited sample and found different prognostic criteria (delirium/polypharmacy). In the absence of comparison with a younger adult population, researchers could not conclude on the existence of specific prognostic criteria for older adults.

Therefore, we conducted a retrospective study on all adults admitted for rhabdomyolysis to Rennes University Hospital between June 2013 and December 2019, to investigate whether factors related to 30-day mortality varied according to patient age.

## Methodology

Single-center retrospective observational study.

### Inclusion

We identified patients over 18 years of age who were admitted to Rennes University Hospital with rhabdomyolysis between June 2013 and December 2019, according to the medico-administrative database of the medicalized information systems program. This database is integrated in a warehouse that provides several data associated with each hospital stay (including emergency visits), such as the main diagnosis (which justified admission) and secondary diagnoses (which were not the main cause of admission, but explained at least part of it). We used the International Classification of Diseases codes (10th edition, ICD10): M62.8 (Rhabdomyolysis) and T79.6 (Traumatic muscle ischemia) to identify the primary and secondary diagnoses of rhabdomyolysis. Patients with CK levels lower than five times the normal range (1000 IU/L) were excluded because they did not meet our definition of rhabdomyolysis [9]. We also excluded adults under temporary legal protection and those in detention. The remaining patients were included and received a letter informing them of their inclusion in this study. Without any objections from them within three weeks, their data were collected. The primary endpoint was 30-day in-hospital mortality.

The study met the standards of the French research regulatory authority’s criteria for observational studies (Declaration date: August 20, 2018. Declaration number: 2,205,295 v 0). All live patients were informed about the study by regular mail, and given the possibility to refuse. The need for informed consent was waived by the ethics committee of Rennes University Hospital (Opinion number: 20.97), due to the low risk design (retrospective observational study) and according to French Law (official journal 2018 − 155 on the processing of personal data in research).

### Collected data

The choice of data to be collected was based on previous studies [8, 10, 11]. We collected demographic data (age, sex, rural or urban lifestyle); presence of professional help at home; Charlson comorbidity index [12, 13]; number of medications (active ingredients) on admission [14]; and weight and initial quick Sequential Organ Failure Assessment (qSOFA) score. This score ranges from 0 to 3, and its components include blood pressure, respiratory rate, and upper function disorders. A ≥ 2 score is associated with a > 10% mortality risk in emergency department patients with a suspected infection [15]. This score was therefore compiled as a categorical variable (< 2 or ≥ 2). We collected the etiology of rhabdomyolysis based on McMahon’s classes: prolonged immobilization, muscular (including myopathy, physical exercise, and sepsis); iatrogenic (statins, neuroleptics), and toxic (cocaine) or other. We also collected biological data from the first 24 h of admission: CK, creatinine, GFR estimated according to the CKD EPI formula [16], hemoglobin, hematocrit, leukocytes, platelets, albumin, blood phosphorus, calcium, bicarbonates and pH. Myoglobinuria data were not available for this retrospective study. We retrieved CK and creatinine maximum concentrations during the stay (named peak concentrations, whatever the length of admission) and recorded the presence of CK over 5000 UI/L, as 5000 UI/L is considered as the threshold from which monitoring and treatment are required [17]. Finally, we recorded the occurrence of complications, such as acute renal failure (assessed according to medical findings when available, or by a 30% decrease in estimated GFR), disseminated intravascular coagulation, compartment syndrome, or delirium, as well as the prescribed intravenous fluid infusion, post-discharge outcome, and 30-day in-hospital mortality.

### Statistical analysis

A univariate analysis was initially performed by comparing the presence and frequency of the different known risk factors by age group. We then looked for an association between each of the data collected and the 30-day in-hospital mortality rate. Statistical significance was set at *p* < 0.05. For categorical variables, we used the uncorrected chi-square test or Fisher exact test when necessary. For continuous variables, when data were > 30 and normally distributed, we used the t-test and parametric Kruskal-Wallis test otherwise. As we found several possible assessment methods for CK (level or peak) and renal function, we chose to proceed to exploratory analysis with both CK peak and CK threshold (> 5000), as well as with creatinine peak and renal failure, as defined in 2.2. The relatively low mean of CK and creatinine levels obtained in our sample drove us to choose the CK peak (instead of a threshold) and the creatinine peak (instead of occurrence of renal failure) for multivariate analysis.

Finally, we performed a multivariate analysis according to the stepwise top-down logistic regression model to identify the different independent prognostic criteria, with an entry threshold *p* < 0.1 in univariate analysis, and an exit threshold *p* > 0.05. In this analysis, we excluded variables with more than 10% missing data (blood phosphorus and calcium). The variables included in the model were age, Charlson score, qSofa score, CK level, peak creatinine level, and presence of disseminated intravascular coagulation or delirium. Epi Info 7 software was used for univariate analysis, and Jamovi software for multivariate analysis.

## Results

**Fig. 1:**
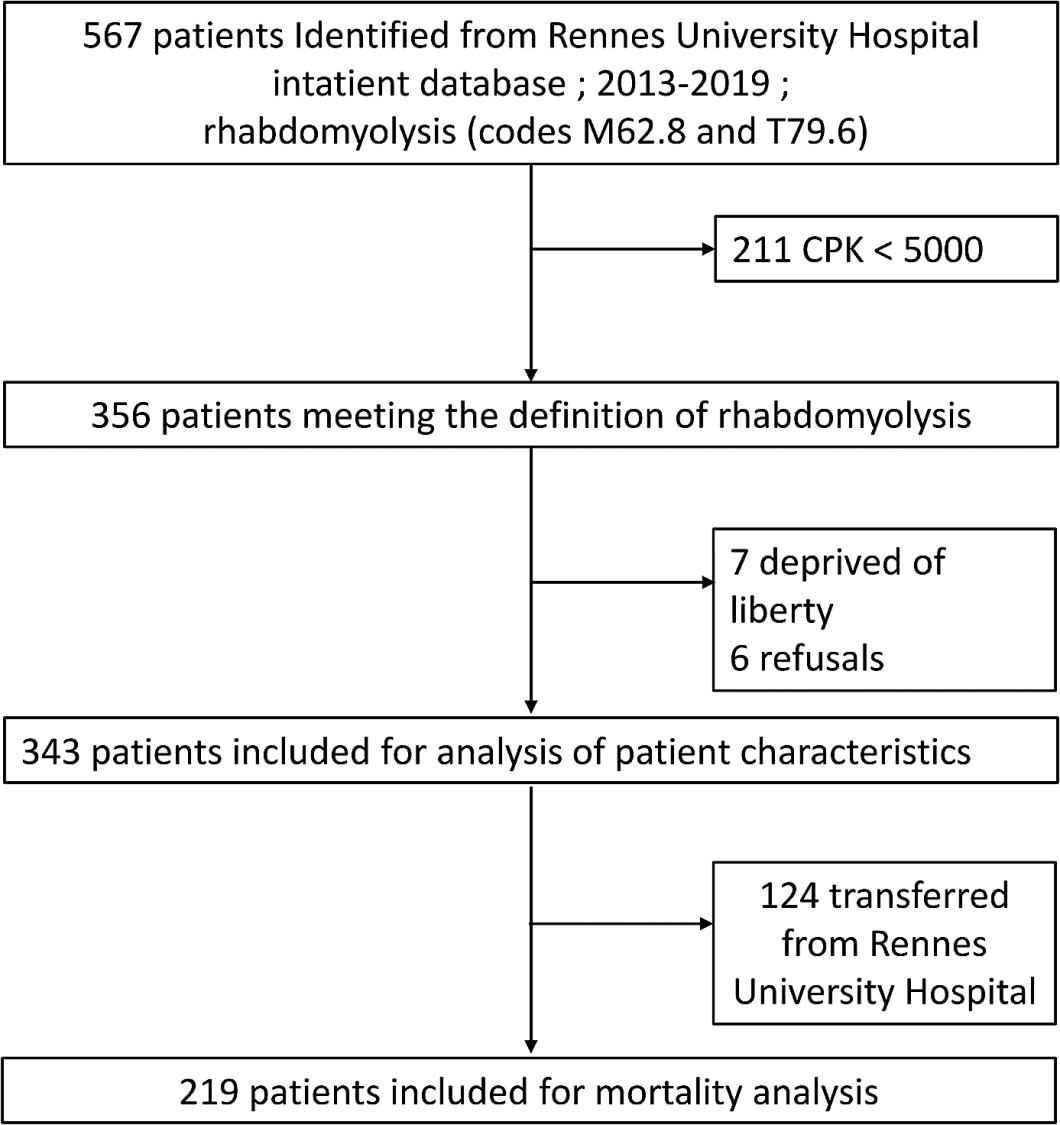
Flow chart

From June 2013 to December 2019, 567 patients aged 18 years or older were admitted to Rennes University Hospital with a primary or secondary diagnosis of “rhabdomyolysis”. Of these patients, 211 had CK levels below 5 times normal. We therefore selected the remaining 356 patients for actual rhabdomyolysis. Seven patients were excluded from the study because they were in detention. We contacted the other patients according to the planned protocol, and six refused to participate. Thus, 343 patients were included in the analysis. Of these, 124 were transferred to another hospital after an initial several-hours to a few-days hospital stay. Therefore, in-hospital mortality and complication rates were only available for 219 patients. [Figure 1 Flow chart]

### Characteristics of the population

The population’s characteristics are summarized in Table 1. The median age was 75 years (range 18–103 years), and 45.2% were women. The population as a whole had a fairly low Charlson comorbidity index (median 1) but was exposed to polypharmacy with a mean of 4.7 active drug ingredients per day (0–19), and 50% taking five or more drugs per day. Prolonged immobilization (65.2% of cases) was the most common etiology of rhabdomyolysis, followed by muscular causes (24.3%), such as seizures, sepsis, and strenuous exercise. The mean CK level on admission was 10,059 IU/L. The mean peak was 21,825 IU/L, and high variability was observed (standard deviation: 195,884 IU/L). The mean phosphorus level was 1.2 mmol/L and the mean potassium level, 4.3 mmol/L. The mean creatinine level on admission was 122.3 µmol/L and the mean peak was 142.3 µmol/L (standard deviation: 149 µmol/L). Acute renal failure was observed in 57.7% of cases, and 25.8% of patients received > 2 L intravenous hydration in the first 24 h of admission. Four patients underwent a renal dialysis. Delirium was observed in 23.8% of the cases. Of included patients,71.2% returned home after hospitalization. We do not know the fate of patients transferred to other hospitals after admission in the emergency ward (35,9%). Finally, we observed 23 in-hospital deaths at 30 days, that is a 10.5% mortality rate. The high number of missing data for phosphorus, calcium, pH, or bicarbonate levels highlights different clinical practices for routine measurement of these parameters.

**Table 1:** Characteristics of patients with rhabdomyolysis: clinical, biological, complications and outcome

Data at inclusion		Total (n = 343)	Missing data
Gender	Female, N (%)	155 (45.2)	0
Age (years)	Mean (SD)	68.7 (20.2)	0
Rural / Urban	Rural, N (%)	64 (18.7)	0
Home help	Yes, N (%)	138 (40.2)	3
Charlson	Mean (SD)	1.3 (1.7)	1
Number of medications	Mean (SD)	4.7 (3.5)	9
qSOFA ≥ 2	Yes, N (%)	30 (8.8)	1
Etiology	Drop, N (%) Drug abuse, N (%) Muscular, N (%) Toxic, N (%) Other, N (%)	223 (65.2) 10 (2.9) 80 (23.3) 22 (6.4) 7 (2.0)	1
Initial CK (IU/L)	Median (25-75%)	2,584 (1,372-6,629)	0
Initial creatinine (µmol/L)	Median (25-75%)	83 (65–124)	2
Potassium (mmol/L)	Median (25-75%)	4.0 (3.7–4.5)	5
Hemoglobin (g/dl)	Median (25-75%)	13.3 (12.2–14.4)	5
Hematocrit (%)	Median (25-75%)	40.0 (36.5–43.4)	5
Platelets (G/L)	Median (25-75%)	220.5 (161–274)	5
Leukocytes (G/L)	Median (25-75%)	11.8 (9.0-14.7)	7
Calcium (mmol/L)	Median (25-75%)	2.2 (2.1–2.4)	150
Phosphorus (mmol/L)	Median (25-75%)	1.0 (0.8–1.3)	174
pH	Median (25-75%)	7.4 (7.3–7.5)	260
Bicarbonates (mmol/L)	Median (25-75%)	23 (20–26)	144
Complications		Total N = 219	
Peak CK (IU/L)	Median (25-75%)	3,288 (1,578-8,491)	0
CK > 5000 UI/L	Yes, N (%)	86 (39.3)	0
Peak creatinine (µmol/L)	Median (25-75%)	91 (69–136)	1
Acute renal failure	Yes, N (%)	124 (56.6)	4
Disseminated intravascular coagulation	Yes, N (%)	4 (1.8)	5
Compartment syndrome	Yes, N (%)	7 (3.2)	5
Delirium	Yes, N (%)	51 (23.3)	5
Treatment			
Infusion	No, N (%) < 1 L, N (%) 1–2 L, N (%) > 2 L, N (%)	18 (8.2) 66 (30.1) 70 (33.5) 55 (25.1)	6
Renal dialysis	N (%)	4 (1.8)	
Outcomes		N = 219	
Release	Return home, N (%) Post Acute Care, N (%) Nursing Home, N (%) Deaths, N (%)	156 (71.2) 35 (16.0) 5 (2.3) 25 (11.4)	0
30-days hospital mortality	Yes, N (%)	23 (10.5)	1

N stands for number; SD stands for standard deviation; qSOFA stands for Quick Sequential Organ Failure Assessment [15];

### Associations between prognostic factors and age categories

The relationship between the presence of known prognostic factors for mortality in rhabdomyolysis and patient age (sorted into 10-year categories) is summarized in Table 2. The etiology of rhabdomyolysis varied with age: 90% of cases in patients aged 70 and over were explained by prolonged immobilization. The Charlson score varied significantly with age and was higher in patients aged 60–69 years (*p* < 0.001). The number of medications was also associated with age, with a maximum of 6.1 per day in the 70–79 age group (*p* < 0.001). The initial and peak CK levels were significantly higher in younger patients (*p* < 0.001), with a 80,534 IU/L mean peak CK in under 50-year-old patients, versus 3,173 IU/L in those over 90 years of age. Peak creatinine was also significantly higher in younger patients, with a mean of 201.4 µmol/L in patients under 50, and 100.7 µmol/L in those over 90 (*p* < 0.001). In contrast, a ≥ 2 qSOFA score on admission to the emergency department was not significantly associated with age (*p* = 0.4). We found no significant difference in admission phosphorus levels according to age (*p* = 0.2). The occurrence of complications such as acute renal failure (*p* = 0.6), delirium (*p* = 0.2), disseminated intravascular coagulation (*p* = 0.1), and 30-day in-hospital mortality (*p* = 0.1) was not associated with age.

**Table 2:** Association between known prognostic factors and age categories according to the Kruskal-Wallis test

	< 50 years n = 64	50–59 years n = 43	60–69 years n = 43	70–79 years n = 48	80–89 years n = 105	> 90 years n = 40	*P* N = 343
Charlson mean(SD)	0.3 (0.8)	1.8 (2.4)	1.9 (2.3)	1.5 (2.0)	1.3 (1.2)	1.3 (1.5)	< 0.001
Number of drugs mean(SD)	2.7 (3.3)	3.6 (3.0)	5.4 (3.3)	6.1 (4.0)	5.4 (3.0)	5.1 (3.0)	< 0.001
Drugs < 8 N(%)	37 (57.8)	19 (44.2)	9 (22.5)	11 (24.9)	13 (12.9)	7 (17.5)	< 0.001
qSOFA ≥ 2 N(%)	8 (12.7)	6 (14.3)	3 (7.0)	3 (6.3)	9 (8.6)	1 (3.3)	0.38
Prolonged immobilization N(%)	5 (7.9)	17 (39.5)	22 (51.2)	43 (89.6)	98 (93.3)	39 (97.5)	< 0.001
Initial CK (median)	3,150 (1,331 − 13,902)	4,507 (1,952 − 10,502)	3,959 (1,560–7,691)	3,528 (1,495–6,128)	1,830 (1,316–4,405)	1,931 (1,280–3,636)	< 0.001
CK peak (median)	5,988 (2,579 − 21,783)	4,754 (1,925 − 12,430)	4,072 (1,990 − 17,595)	4,122 (1,805–8,661)	1,900 (1,340–4,601)	1,993 (1,326–3,636)	< 0.001
CK ≥ 5,000 UI/L N(%)	34 (54.0)	21 (48.8)	16 (37.2)	19 (39.6)	25 (23.8)	5 (12.5)	< 0.001
Peak creatinine (median)	105 (73–200)	91 (68–217)	124 (88–177)	83.5 (70–111)	84 (61–125)	86.5 (69.5–110)	0.01
Phosphorus (median)	1.1 (0.9–1.4)	1.0 (0.8–1.4)	1.1 (0.8–1.4)	0.9 (0.8–1.1)	0.9 (0.8–1.1)	1.1 (0.9–1.4)	0.20
Bicarbonates (median)	24 (18–26)	23 (19–25)	22.5 (20–25)	22 (19–24)	23 (20–25)	23.5 (21–27)	0.62
Acute Renal Failure N(%)	35 (56.5)	26 (61.9)	29 (67.4)	19 (52.9)	40 (50.6)	16 (59.3)	0.55
Dissaminated Intravascular coagulation N	1	3	1	0	0	0	0.09
Delirium N(%)	13 (21)	12 (28.6)	6 (14.3)	9 (25.7)	28 (34.6)	8 (27.6)	0.23
IV infusion > 2 L N(%)	27 (43.6)	13 (33.3)	12 (29.3)	11 (23.9)	14 (13.9)	1(2.6)	< 0.001
Returned home N(%)	48 (76.2)	21 (48.8)	24 (55.8)	15 (31.3)	34 (32.4)	11 (27.5)	< 0.001
30-day hospital mortality N(SD)	1 (1.7)	2 (4.9)	7 (17.1)	4 (8.9)	8 (8.9)	3 (10)	0.14

qSOFA stands for quick Sequential Organ Failure Assessment [15]

SD stands for Standart Deviation

N stands for Number

% stands Percentages, calculated taking into account missing data

### Factors associated with 30-day in-hospital mortality

The univariate analysis between the different variables collected and 30-day in-hospital mortality is summarized in Table 3 for the 219 patients whose in-hospital mortality data were verified. We found the variables significantly associated with mortality were age (73.2 years versus 62.6 years respectively for deceased and surviving patients; *p* < 0.02); Charlson comorbidity score (2.7 vs. 1.1; *p* < 0.05); ≥ 2 qSOFA score at admission (34.8% vs. 6.2%; *p* < 0.001); admission creatinine (223.2 µmol/L vs. 114.9 µmol/L; *p* < 0.001), peak creatinine (282.5 µmol/L vs. 137.3 µmol/L; *p* < 0.001), lowest GFR (25.5 ml/min vs. 66.7 ml/min; *p* < 0.001), development of acute renal failure (91.3% vs. 53.6%; *p* < 0.001), admission phosphorus level (1.9 mmol/L vs. 1.1 mmol/L; *p* < 0.02), occurrence of DIC (13.6% vs. 0.5%; *p* < 0.01) and delirium (45.5% vs. 21.4%; *p* < 0.05). In contrast, baseline CK (20,854 IU/L vs. 11,468 IU/L) and peak CK (23,983 IU/L vs. 30,095 IU/L) did not show a statistically significant association with mortality. Analysis of over-threshold (> 5000 IU/L) CK levels as a categorical variable also showed no significant difference in 30-day in-hospital mortality (56.5% vs. 37.2%).

**Table 3:** Univariate analysis between the different variables and 30-day in-hospital mortality using the T-test for continuous variables and the Chi-2 test for categorical variables (non-transferred patients N = 219)

		Total (N = 219)	Deceased (n = 23)	Living (n = 196)	*P*
Gender	Female, N (%)	83 (37.9)	7 (30.4)	76 (38.8)	0.44
Age (years)	Mean (SD)	63.7 (21.2)	73.2(17.0)	62.6 (21.4)	**0.013**
Rural / Urban	Rural, N (%)	44(20.1)	4 (17.4)	40 (20.4)	1.0
Support	Yes, N (%)	74 (34.1)	4 (19.1)	70 (35.7)	0.13
Charlson	Mean (SD)	1.2 (19)	2.7 (3.6)	1.1 (1.5)	**0.046**
Number of medicines	Mean (SD)	4.2 (3.4)	4.2 (3.2)	4.2 (3.4)	0.97
qSOFA ≥ 2	Yes, N (%)	20 (9.2)	8 (34.8)	12(6.2)	**0.001**
Etiology	Drop, N (%) Drug abuse, N (%) Muscular, N (%) Toxic, N (%) Other, N (%)	117 (53.4)	14 (60.1)	103 (52.6)	
Initial CK (IU/L)	Median (25-75%)	7 (3.2)	0 (0.0)	7 (3.6)	0.26
Peak CK (IU/L)	Median (25-75%)	73 (33.3)	8 (34.8)	65 (33.2)	0.76
CK 5000 (UI/L)	Yes, N (%)	15 (5.0)	0 (0.0)	15 (6.9)	0.11
Peak creatinine (µmol/L)	Median (25-75%)	7 (3.2)	1 (4.3)	6 (3.1)	**0.001**
Acute renal failure	Yes, N (%)	2,818 (1,438–7,400)	5,903 (2,075 − 17,593)	2,705 (1,370–6,491)	**0.001**
Potassium (mmol/L)	Median (25-75%)	3,579 (1,665–9,425)	5,957 (2,166 − 17,595)	3,528 (1,641–8,115)	0.12
Hemoglobin (g/dl)	Median (25-75%)	86 (39.3)	13 (56.5)	73 (37.2)	0.46
Hematocrit	Median (25-75%)	92 (70–153)	301 (159–405)	89 (68–130)	0.88
Platelets (G/L)	Median (25-75%)	124 (57.7)	21 (91.3)	107(53.6)	0.32
Leukocytes (G/L)	Median (25-75%)	4.1 (3.6–4.6)	4.6 (3.9–5.2)	4.0 (3.6–4.5)	0.61
Calcium (mmol/L)	Median (25-75%)	13.4 (12.2–14.7)	12.5 (10.5–15.5)	13.4 (12.3–14.5)	0.052
Phosphorus (mmol/L)	Median (25-75%)	40.3 (36.3–44.0)	38.6 (33.2–47.1)	40.4 (36.3–43.8)	**0.015**
pH	Median (25-75%)	217 (159–274)	190 (102–257)	218 (161–276.5)	0.45
Bicarbonates (mmol/L)	Median (25-75%)	11.6 (8.6–14.5)	12.3 (8.1–18.8)	11.4 (8.6–14.2)	0.11
DIC	Yes, N (%)	2.2 (2.1–2.4)	1.9 (1.7–2.2)	2.2 (2.1–2.4)	**0.003**
Compartment syndrome	Yes, N (%)	1.0 (0.8–1.4)	2.0 (1.4–2.5)	1.0 (0.8–1.3)	0.15
Delirium	Yes, N (%)	7.4 (7.3–7.5)	7.3 (7.3–7.4)	7.4 (7.3–7.5)	**0.012**
Intravenous hydration	No, N (%) < 1 L, N (%) 1–2 L, N (%) > 2 L, N (%) Dialysis, N (%)	22 (18.5–26)	18 (14–22)	23 (20–26)	
Hydration > 2 L	Yes, N (%)	4 (1.9)	3 (13.6)	1 (0.5)	**0.008**

N stands for number; SD stands for standard deviation, qSOFA stands for quick Sequential Organ Failure Assessment [15];

%stands for percentages, calculated taking into account missing data

### Multivariate analysis

To identify independent prognostic variables, we performed a multivariate analysis of in-hospital 30-day mortality according to variables that were statistically significant in the univariate analysis (Table 4). Five variables were identified: age (*p* = 0.003); Charlson score (*p* = 0.015); initial qSOFA score (*p* = 0.002); peak creatinine (*p* = 0.002) and the existence of DIC (*p* = 0.011).

**Table 4:** Variables with less than 10% missing data associated with 30-day in-hospital mortality in rhabdomyolysis, according to the logistic regression model (non-transferred patients N = 219)

Variables	Odds ratio	95% CI	*p*
Intercept	2.47e-4	[7.25e-6; 0.008]	< 0.001
Age	1.06	[1.02; 1.10]	0.003
Charlson	1.30	[1.05; 1.60]	0.015
qSOFA	5.11	[1.31; 19.98]	0.002
Peak creatinine	1.00	[1.00; 1.01]	0.002
Disseminated intravascular coagulation	37.68	[2.29; 620.59]	0.011

Accuracy: 0.919

The variables included in the model were: age, Charlson score, qSofa score, CK level, peak creatinine level and presence of Disseminated intravascular coagulation or delirium

## Discussion

In this study, we sought to identify whether there existed an age difference in the factors related to 30-day in-hospital mortality in rhabdomyolysis. We observed that specific known prognostic factors [8–11], such as sex, CK and creatinine levels on admission, lower estimated GFR, or number of medications, were not randomly distributed according to age. We therefore demonstrated that age influences the patients’ prognostic profile. Our population’s median age was 75.0 years– much older than the populations tested to develop prognostic scores (50.7 years in McMahon, 40.0 years in Simpsons), and more representative of a geriatric population.

In univariate and multivariate analysis, age proved to be a factor not proportionally associated with 30-day in-hospital mortality after rhabdomyolysis, with increased mortality among the 60–69 age group. Other factors associated with mortality were the Charlson comorbidity score, initial qSOFA score, and peak creatinine level. Our results did not show an association between phosphorus levels and mortality, but such a link cannot be excluded, considering the possible impact of missing data on statistical power. Phosphorus level at admission was included in the Mc Mahon risk prediction score for kidney failure or mortality validated in a younger cohort [8], and was associated with renal failure but not with mortality in the Wongrakpanich older cohort [10]. Further studies would be necessary to document the role of phosphorus measurements for prognostic purposes in older rhabdomyolysis patients. The number of medications at admission did not come out as a prognostic criterion, contrary to what Wongrakpanich described in an exclusively older population (range: 66–101, median: 80 years) [10]. In our study, age was significantly associated with the number of drugs, with a maximum number of drugs observed in the 70–79 age group (i.e., at the median patients age in the Wongrakpanich study). This finding tends to validate the hypothesis that age influences the prognostic factors linked to rhabdomyolysis, notably through multimorbidity.

One of the limitations of our study was the amount of missing data, due to the retrospective design. Mortality after hospital discharge was not available in our warehouse. Therefore, we chose 30-day in-hospital mortality for primary endpoint. Although this endpoint may underestimate mortality, McMahon [8] and Simpson [9] used it to develop and validate a predictive score in rhabdomyolysis. Indeed, 124 patients with rhabdomyolysis were transferred to local hospitals, and we were therefore unable to collect the biological evolution, complications, and in-hospital mortality at 30 days. Local hospitals offer geriatric units, with no intensive care unit and no computed tomography on site. Transferred patients, which have been distanced from the main hospital’s technical facilities, may be older, present a less severe acute illness, or have a limitation of care order. These transfers led to an attrition bias, whose impact is difficult to estimate. Furthermore, we did not have data to ensure rhabdomyolysis coding exhaustivity in the database we used. However, as the database we analyzed is used by the health insurance for pricing hospital stays, its quality is regularly controlled. As rhabdomyolysis pathology justifies high invoicing, it seems unlikely that a significant number of stays are missing.

The CK level (initial or peak), which is representative of damaged muscle mass, does not prove to be a prognostic criterion for mortality. This finding is in keeping with previous studies [8, 10, 11]. The monitoring of myoglobinuria, an earlier reflection of rhabdomyolysis that is directly related to the occurrence of acute renal failure, could have been relevant, but was not available in this retrospective study [18, 19]. CK levels and peaks are significantly higher in young subjects, probably in relation to muscle mass [20, 21]. Our exploratory analysis argues in favor of defining CK thresholds that may be lower for older adults to retain the diagnosis, assess its severity, and guide the therapeutic strategy.

In multivariate analysis, neither the occurrence of acute renal failure (48.0%) nor peak creatinine level seemed to be associated with 30-day in-hospital mortality, unlike in previous studies. The definition of renal failure we use (30% decrease in estimated GFR) is not consensual [22] but has the advantages of simplicity and availability of measurements. However, this definition carries the risk of misclassifying subjects. Indeed, some older patients may have a particularly low baseline creatinine level in relation to reduced muscle mass, and acute renal failure may therefore go undetected [23]. The frequency of acute renal failure during rhabdomyolysis is highly variable in the literature, ranging from 10 to 58%, depending on the study [3, 4, 8]. Variability can be due to inclusion criteria (CK level, resuscitation), different etiologies, and diagnostic criteria for acute renal failure in these studies [3, 24].

Finally, we found in our study different levels of prescribed fluid infusion according to age. This may be related to different etiologies (septic and muscular origins being more frequent in young subjects), but also to clinicians’ reluctance to use volume expansion in older patients, particularly in cases of cardiac comorbidities. Analysis of the literature shows lower filling levels in older patients, particularly in cases of septic shock, accounted for by the existence of comorbidities [25, 26].

We observed a 10.5% mortality rate, that is 23 deaths, which is slightly lower than rates found in the literature (14.0% in McMahon’s study [8]; 14.0% in the Rodriguez study [3]) but comparable to the Wongrakpanich study’s rates [10] ((10.2%). This may be due to our inclusion criteria (CK > 1000 IU/L vs. 5000 IU/L in many studies). Given the low number of events, a larger study would be useful to confirm these results.

## Conclusion

In our study, the prognostic factors of rhabdomyolysis were not randomly distributed according to age. In view of the decrease in muscle mass with age, the CK and creatinine thresholds usually used in prognostic scores may be questioned: the 5000 IU/L threshold may need to be verified in an older population. The impact of phosphorus and polypharmacy requires further studies on larger samples. Our results highlight age-related clinical and prognostic specificities of patients with rhabdomyolysis, which point to the necessity of further studies in the geriatric population concerning diagnosis, prognostic criteria, and ultimately therapeutic approaches of this pathology, which is quite frequent in old age.

## Data Availability

The datasets used and/or analyzed during the current study are available from the corresponding author on reasonable request.
